# Comparison of inpatient charges and costs between revision and primary total elbow arthroplasty in the New York state

**DOI:** 10.1016/j.xrrt.2025.100648

**Published:** 2025-12-24

**Authors:** Dashaun A. Ragland, Brian O. Molokwu, Jacquelyn J. Xu, Andrew J. Cecora, Sallie Yassin, Erel Ben-Ari, Joseph A. Bosco, Mandeep S. Virk

**Affiliations:** a Division of Shoulder and Elbow Surgery, Department of Orthopedic Surgery, NYU Grossman School of Medicine, NYU Langone Orthopedic Hospital, NYU Langone Health, New York, NY, USA; b Division of Biostatistics, Department of Population Health, NYU Grossman School of Medicine, NYU Langone Health, New York, NY, USA

**Keywords:** Cost, Charges, Economic burden, Elbow, Revision, Arthroplasty

## Abstract

**Background:**

The primary aim of this study is to evaluate differences in inpatient charges between primary (pTEA) and revision (rTEA) total elbow arthroplasty among Medicare and Medicaid patients. Our secondary aim is to assess whether these charges vary across hospitals with differing total elbow arthroplasty (TEA) procedural volumes. We hypothesize that rTEA would be more expensive than pTEA and that charges would be higher for low-volume hospitals.

**Methods:**

The Statewide Planning and Research Cooperative System database was queried for all Medicare and Medicaid Services patients who underwent a pTEA or rTEA in New York State from 2010 to 2020. Hospitals were classified as high-volume (≥3 surgeries/year), medium-volume (between 2-3 surgeries/year), or low-volume (less than 2 surgeries/year). Facilities performing fewer than 1 surgery per year or with fewer than 4 years of TEA data were excluded. Total inpatient charges were collected and subsequently subdivided into ancillary and accommodation charges. Inpatient charges and readmission data were compared across the 2 procedures and volume groups.

**Results:**

During the study period, 1,303 patients underwent pTEA and 273 underwent rTEA. After adjusting for patient age, sex, race, and hospital volume, rTEA was independently associated with significantly higher accommodation, ancillary, and total inpatient charges (*P* < .001 for all). Additionally, rTEA patients had a higher likelihood of 90-day readmission (*P* = .005) and longer inpatient stays (*P* < .001) compared to pTEA patients. There were observable differences in total, accommodation, and ancillary charges across hospital volume groups for both pTEA and rTEA. Low-volume hospitals demonstrated the highest total charges for pTEA during the study period vs. high- and medium- volume hospitals (*P* < .001 for pTEA, *P* > .05 for rTEA).

**Conclusion:**

rTEA is associated with longer inpatient stay, higher inpatient charges, and greater readmission rates compared to pTEA. Primary TEA in low-volume hospitals is associated with higher total charges compared to medium and high-volume hospitals. These findings provide valuable insights for hospital administrators and public health officials aiming to create effective strategies to manage costs and combat the growing burden of healthcare expenses in the United States.

Total elbow arthroplasty (TEA) was initially developed as a treatment for end stage elbow inflammatory arthropathy, but indications have expanded over years to include various pathologies including nonreconstructable acute fractures and fracture sequelae.^20^ TEAs have lower revision-free survivorship compared to hip and knee arthroplasties, and only about 10% of these procedures are performed by fellowship-trained surgeons.^11^^,^^16^^,^^17^ Since projection studies predict an increasing prevalence of primary TEA (pTEA) and revision TEA (rTEA) in the coming years, it is important to understand the financial trends of this procedure as policy makers have placed increased attention on maximizing healthcare value.^5^^,^^18^^,^^21^

Few studies have examined the cost or charges of TEA. Zhou et al^21^ found the mean direct charges of pTEA to be $16,300 ± 4,000 per case at academic institutions, though they recognized the results were limited by the overall low patient volume. Federer et al^10^ concluded that TEA is a slightly more cost-effective procedure than open reduction internal fixation for most elderly patients with distal humerus fractures. To our knowledge, no studies to date have compared the overall charges of pTEA and rTEA within one patient population, nor the impact of the hospital procedural volume on inpatient charges. Our study's primary aim is to determine if there is a difference in inpatient charges between pTEA and rTEA in Medicare and Medicaid patients using the Statewide Planning and Research Cooperative System (SPARCS) database. Our secondary aim is to investigate how these values differ across hospitals with varying TEA procedural volumes. We hypothesize that rTEA will be more expensive than pTEA and that overall charges will be higher for low-volume hospitals.

## Materials and methods

### Data collection

The SPARCS database was queried for all patients who underwent a pTEA or rTEA in New York State from January 2010 to December 2020. This query was based on an inclusive list of procedural International Classification of Diseases, Ninth (ICD-9) and 10th Revision (ICD-10), and Current Procedural Terminology (CPT) codes. The SPARCS database is a detailed reporting system managed by the Department of Health, collecting all preadjudicated inpatient and outpatient claims across New York State.^6^ It offers patient-level data on demographics, admission diagnoses, treatments, and hospital charges. Because trends identified from SPARCS data have generally resembled those of provider utilization and payment data provided by the Centers for Medicare & Medicaid Services, various other studies have utilized SPARCS to investigate total joint arthroplasty trends and associated hospital charges.^8^^,^^15^^,^^19^ The primary study outcomes were ancillary, accommodation, and total inpatient charges for each pTEA and rTEA hospital encounter, reported in current US dollars. Secondary outcomes included the impact of hospital TEA volume on inpatient charges and 90-day readmission characteristics for pTEA and rTEA overall, as well as when stratified by hospital volume groups. To estimate the association between surgery type and inpatient charges and readmission rates, multivariable regression analyses were also performed, adjusting for patient age, sex, race, and hospital surgical volume.

### “Charges” vs. “costs”

The terms "charges" and "costs" have distinct definitions. According to the American Medical Association, ‘costs’ represent the amount a patient pays for services rendered, while ‘charges’ represent the amount asked by a provider for a healthcare good or service, which appears on a medical bill.^3^ Total inpatient charges were subdivided into ancillary and accommodation charges to provide a more detailed understanding of charge allocation per hospital encounter.^15^ The Centers for Medicare & Medicaid Services define ancillary charges (ANCIL) as services pertaining to laboratory, radiology, pharmacy, perioperative, operative, therapy, implant, and special items services.^1^^,^^15^ Accommodation charges (ACCOM) include fees associated with patient room and board. The total charges (TOT) are roughly equal to the sum of the ANCIL and ACCOM. Total ‘costs’ for pTEA and rTEA throughout the study period were also recorded.

### Generation of study groups

pTEA patients were identified using CPT and ICD-9 and ICD-10 procedure codes. Specific diagnoses included nonunion, distal humerus fracture, osteoarthritis, stiffness/contracture, inflammatory arthropathy, deformity, and articular cartilage disorders. To identify rTEA patients, a list of relevant postoperative indications for revision procedures was compiled based on the revision ICD and CPT codes. Patient demographics, diagnosis codes, procedure volumes, and charges were collected. Additional outcomes collected included perioperative variables such as length of stay (LOS) and discharge location.

### Hospital volume

Because pTEA and by proxy rTEA are relatively rare joint replacement procedures,^21^ hospitals in the SPARCS database were categorized into 3 volume-based groups: high, medium, and low. These groups were defined based on the consistency and distribution of TEA volume over the 10-year study period. High-volume hospitals (HVHs) performed 3 or more TEAs per year, medium-volume hospitals (MVHs) performed between 2 to 3 surgeries per year, and low-volume hospitals (LVHs) performed less than 2 TEAs per year. Hospitals with fewer than 1 TEA performed per year, or less than 4 active years (ie a year where ≥1 TEAs were performed) were excluded. In total, 19 hospitals were categorized as HVH, 24 as MVH, and 33 as LVH based on these criteria.

### Complications and revisions

To assess and compare the impact of indication on charges for rTEA, reasons for revision surgery were initially categorized into several subcategories based on specific clinical indications. However, many of these subcategories had small sample sizes, which limited the statistical power and interpretability of the analysis. To address this, we consolidated the indications into 2 broader categories: mechanical complications (eg, aseptic loosening, instability) and nonmechanical complications (eg, infection, periprosthetic fracture). This approach allowed us to conduct a more clinically meaningful analysis while maintaining sufficient sample sizes for robust statistical comparison.

Complications for both pTEA and rTEA were evaluated by treating each procedure type as its own index admission and identifying any subsequent admissions within 90 days of that index event. Readmissions for any reason within this period were classified as adverse outcomes. Consequently, our analysis included both implant-specific and non–implant-specific complications, such as infections of any origin, myocardial infarction, pulmonary embolism, and inhospital mortality.

### Statistical analysis

Simple comparisons between the groups were performed with standard statistical tests (ie, t tests, and χ2 tests as appropriate). Specific variables, such as LOS and charges, were not normally distributed but were assumed to have similar distributions; therefore, Mann–Whitney U tests were utilized to compare distributions of these data between pTEA and rTEA. Mann–Whitney U test results are typically reported using median and interquartile range rather than mean and standard deviation, as it is a nonparametric test. A bivariate analysis was performed to assess differences in readmission characteristics (rate of readmissions, time from surgery to readmission (days), the number of times readmission occurred within ninety days, and LOS) between HVHs, MVHs, and LVHs. For regression analyses, model estimates are presented as β coefficients with 95% confidence intervals (CIs) for linear regressions and log-odds coefficients/odds ratios (ORs) with 95% CIs for logistic regression. All tests were performed with a statistical significance set at *P* < .05.

## Results

### Patient cohort

Thirty-eight hospitals (collectively performing 128 TEAs during the study period) were excluded from the analysis due to each of these centers having fewer than 1 TEA performed per year, or less than 4 active years. The final cohort consisted of 1,576 surgeries with 273 (17.3%) undergoing a rTEA and 1,303 (82.7%) undergoing a pTEA. The average age of the cohort was 60.4 years, with 66.1% (1,042) female patients (Table I).

**Table I:** Differences in demographic and clinical characteristics between primary and revision TEA from 2010-2020.

Variable	Overall (n = 1,576)	Primary TEA (n = 1,303)	Revision TEA (n = 273)	*P* value
Age (yr) (mean ± SD)	60.4 ± 17.0	61.3 ± 17.1	56.4 ± 15.9	**<.001**∗
Gender (%)				**<.001**†
Female (F)	1,042 (66.1)	892 (68.5)	150 (54.9)	
Male (M)	534 (33.9)	411 (31.5)	123 (45.1)	
Race (%)				**.04**†
Black/African American	94 (7.2)	80 (7.5)	14 (5.9)	
Other	255 (19.5)	221 (20.7)	34 (14.2)	
White	957 (73.3)	766 (71.8)	191 (79.9)	
Length of stay (d)				**.001**‡
Median [IQR]	3.00 [2.00, 6.00]	3.00 [2.00, 5.00]	4.00 [2.00, 6.00]	
Discharge location (%)				**<.001**†
Home	959 (61.0)	807 (62.0)	152 (55.9)	
Home with services	309 (19.6)	238 (18.3)	71 (26.1)	
Nonhome	76 (4.8)	55 (4.2)	21 (7.7)	
Skilled nursing facility	229 (14.6)	201 (15.4)	28 (10.3)	

*TEA*, total elbow arthroplasty; *IQR*, interquartile range; *SD*, standard deviation.

Bolded *P* values denote a statistically significant value.

∗ Student's t-test.

† Pearson chi-squared test.

‡ Mann–Whitney U test.

Nineteen hospitals were classified as HVH (performing 863 TEAs collectively). The MVH group consisted of 24 hospitals (performing 405 TEAs collectively), while the LVH group consisted of 33 hospitals (performing 308 TEAs collectively). Differences in demographic and clinical characteristics between different hospital volume groups are shown in Table II.

**Table II:** Differences in demographic and clinical characteristics between hospital TEA volumes from 2010-2020.

Variable	Overall (n = 1,576)	High (n = 863)	Medium (n = 405)	Low (n = 308)	*P* value
Age (yr) (mean ± SD)	60.4 ± 17.0	59.9 ± 16.5	59.4 ± 17.6	63.3 ± 17.2	**.003**∗
Gender (%)					.30†
Female (F)	1,042 (66.1)	585 (67.8)	261 (64.4)	196 (63.6)	
Male (M)	534 (33.9)	278 (32.2)	144 (35.6)	112 (36.4)	
Race (%)					**.02**†
Black/African American	94 (7.2)	39 (5.6)	28 (7.8)	27 (11.0)	
Other	255 (19.5)	146 (20.8)	74 (20.6)	35 (14.3)	
White	957 (73.3)	516 (73.6)	258 (71.7)	183 (74.7)	
Length of Stay (d)					.13‡
Median [IQR]	3.00 [2.00, 6.00]	3.00 [2.00, 5.00]	3.00 [2.00, 5.00]	3.00 [2.00, 6.00]	
Discharge Location (%)					**.03**†
Home	959 (61.0)	530 (61.5)	251 (62.1)	178 (58.0)	
Home with services	309 (19.6)	184 (21.3)	65 (16.1)	60 (19.5)	
Nonhome	76 (4.8)	41 (4.8)	15 (3.7)	20 (6.5)	
Skilled nursing facility	229 (14.6)	107 (12.4)	73 (18.1)	49 (16.0)	

*TEA*, total elbow arthroplasty; *IQR*, interquartile range; *SD*, standard deviation.

Bolded *P* values denote a statistically significant value. “n” refers to the number of total elbow arthroplasty cases performed collectively amongst hospitals in the volume group.

∗ Student's t-test.

† Pearson chi-squared test.

‡ Mann–Whitney U test.

### Clinical disposition characteristics

Median LOS for pTEA was 3 days compared to 4 days for rTEA (*P* < .001). Nine hundred fifty-nine (61.0%) patients in the total cohort were discharged home without additional caregiving services, while 229 (14.6%) were discharged to a skilled nursing facility. Eight hundred seven (62.0%) of the pTEA patients were discharged home with no additional services compared to 152 (55.9%) of the rTEA cohort (*P* < .001) (Table I). The differences in discharge disposition for pTEA and rTEA patients across different hospital volume groups are shown in Table II.

### Comparison of charges and costs between primary and revision total elbow arthroplasty

Bivariate analyses demonstrated no significant differences in ACCOM, ANCIL, and TOT charges between pTEA and rTEA (Supplemental Table I). However, after adjusting for patient age, sex, race, and hospital volume in a multivariate linear regression model, rTEA was independently associated with significantly higher ACCOM, ANCIL, and TOT charges compared with pTEA (*P* < .001 for all) (Table III).

**Table III:** Multivariable linear regression analysis of inpatient charges for revision vs. primary TEA in US dollars, adjusted for patient age, gender, hospital surgical volume, and race.

Variable	β coefficient (US Dollars)∗†	95% CI	*P* value
Accommodation charges	8,009.74	3,469.83-12,549.66	**<.001**
Ancillary charges	11,924.07	5,528.13-18,320.02	**<.001**
Total charges	19,933.81	10,690.26-29,177.37	**<.001**

*TEA*, total elbow arthroplasty; *CI*, confidence interval.

Bolded *P* values denote a statistically significant value. Hospital surgical volume was categorized into low, medium, and high groups based on tertiles of annual TEA case volume.

∗ Positive β values indicate higher adjusted charges for Revision TEA relative to Primary TEA (reference).

† Primary TEA was used as the reference group.

**Figure 1:**
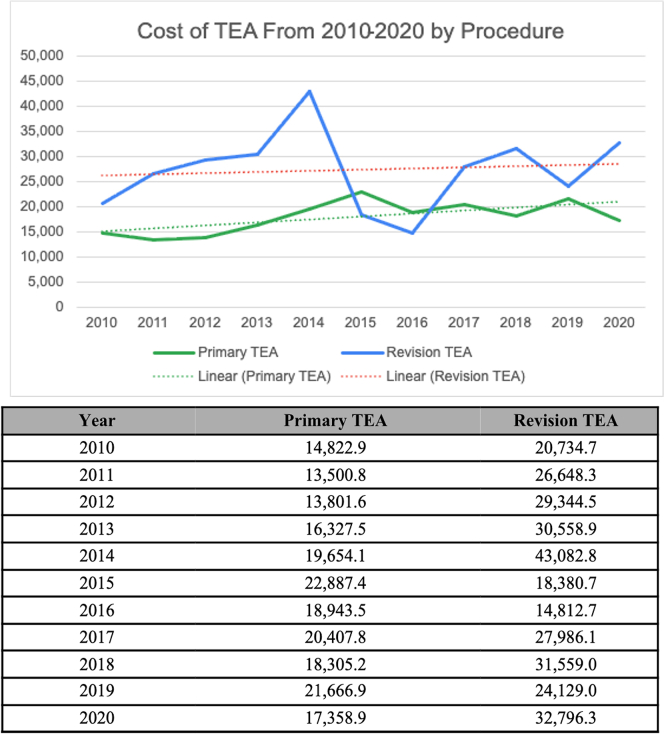
Total costs of primary and revision TEA over time in US dollars. (**A**) Line graph of mean total costs by year for each type of elbow replacement in US dollars. (**B**) Corresponding table of average total costs for each total elbow replacement. *TEA*, total elbow arthroplasty.

From 2010 to 2020, the average cost (ie, the actual amount a patient pays) of pTEA increased from $14,823 to $17,359 and rTEA costs increased from $20,735 to $32,796 over the study period (Figure 1).

### Comparison of charges and costs of total elbow arthroplasty across different hospital volumes

**Figure 2:**
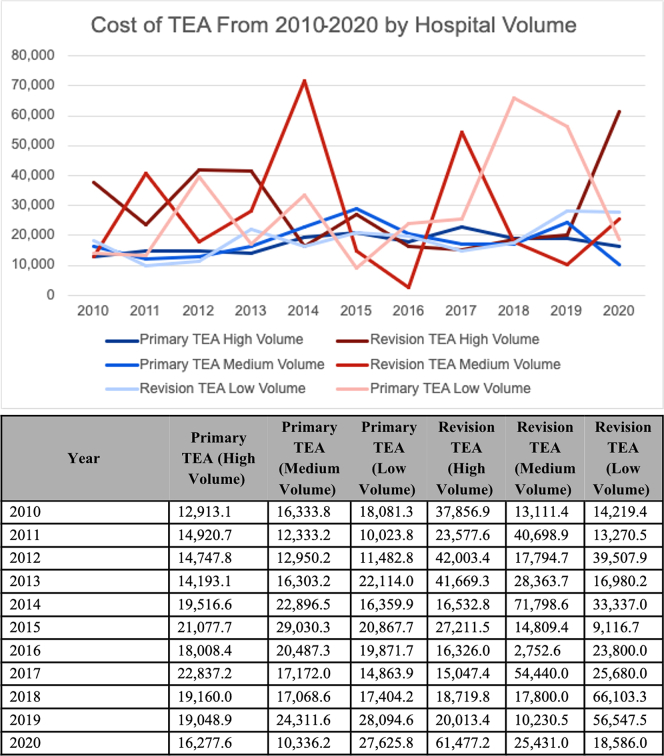
Total costs of primary and revision TEA over time stratified by hospital volume in US Dollars. (**A**) Line graph of mean total costs by year for each type of elbow replacement, stratified by hospital TEA volume, in US dollars. (**B**) Corresponding table of annual mean total costs for each total elbow replacement stratified by hospital TEA volume. *TEA*, total elbow arthroplasty.

LVHs showed higher median TOT ($61,857) for pTEA compared to HVHs ($53,661) and MVHs ($48,584) (*P* < .001). There were no differences in TOT charges for rTEA among different volume hospitals. The breakdown of TOT, ANCIL, and ACCOM charges for different volume hospitals for pTEA and rTEA is shown in Table IV. Cost trends for pTEA and rTEA across hospital volume groups over the study period are shown in Figure 2.

**Table IV:** Analysis of effect of hospital volume and primary/revision TEA on inpatient charges in US Dollars.

Primary TEA	Overall (n = 1,303)	High (n = 755)	Medium (n = 302)	Low (n = 246)	*P* value
Accommodation Charges (Median [IQR])	10,200.00 [5,278.00, 19,871.64]	10,700.00 [5,998.00, 19,510.00]	8,630.00 [4,507.50, 19,703.50]	9,651.00 [4,677.50, 21,638.00]	**.04**∗
Ancillary Charges (Median [IQR])	40,383.30 [27,062.89, 59,728.30]	40,100.05 [28,836.31, 59,622.26]	38,608.85 [20,795.82, 55,041.09]	43,459.65 [24,891.52, 68,314.13]	**.002**∗
Total Charges (Median [IQR])	52,937.13 [36,250.35, 82,410.64]	53,661.00 [38,244.61, 84,000.61]	48,583.85 [29,781.75, 72,374.33]	61,856.84 [34,906.62, 91,961.98]	**<.001**∗

Revision TEA	Overall (n = 273)	High (n = 108)	Medium (n = 103)	Low (n = 62)	*P* value
Accommodation Charges (Median [IQR])	12,650.00 [4,700.00, 25,680.00]	15,275.00 [7,062.50, 30,546.25]	9,500.00 [3,456.00, 20,286.50]	12,866.38 [4,131.00, 24,585.75]	**.03**∗
Ancillary Charges (Median [IQR])	40,663.07 [21,125.55, 76,137.00]	40,409.24 [22,050.62, 78,440.80]	35,842.21 [17,647.30, 70,261.07]	47,888.79 [24,044.88, 75,820.85]	.27∗
Total Charges (Median [IQR])	56,110.73 [28,865.55, 101,748.35]	60,851.90 [33,775.93, 117,640.21]	46,046.52 [25,383.95, 87,300.33]	65,081.56 [32,423.43, 100,182.07]	.13∗

*TEA*, total elbow arthroplasty; *IQR*, interquartile range. Bolded *P* values denote a statistically significant value. “n” refers to the number of total elbow arthroplasty cases performed collectively amongst hospitals in the volume group.

∗ Mann–Whitney U test.

### Impact of indication for revision total elbow arthroplasty on charges

Of the 273 rTEAs in our analysis, 182 were indicated due to a mechanical complication, and 91 were due to a nonmechanical complication. There were no differences in the median ACCOM, ANCIL, and TOT charges for rTEA due to mechanical compared with nonmechanical complications (*P* = .06, *P* = .44, and *P* = .24, respectively) (Table V).

**Table V:** Revision TEA subgroup analysis of effect of indication on inpatient charges in US dollars.

Variable	Overall (n = 273)	Mechanical complication (n = 182)	Nonmechanical complication (n = 91)	*P* Value
Accommodation charges (Median [IQR])	12,650.00 [4,700.00, 25,680.00]	17,571.80 [8,846.00, 31,369.58]	12,472.00 [4,032.00, 17,975.00]	.06∗
Ancillary charges (Median [IQR])	40,663.07 [21,125.55, 76,137.00]	40,228.52 [27,890.05, 63,250.80]	33,688.62 [19,756.83, 66,436.05]	.44∗
Total charges (Median [IQR])	56,110.73 [28,865.55, 101,748.35]	64,497.05 [37,625.06, 102,010.52]	50,492.10 [22,690.82, 83,165.02]	.24∗

*TEA*, total elbow arthroplasty; *IQR*, interquartile range.

Bolded *P* values denote a statistically significant value.

∗ Mann–Whitney U test.

### Readmission characteristics

A total of 170 patients in the cohort were readmitted within 90 days of their respective index surgery (pTEA or rTEA). The rTEA index group was readmitted at a higher rate than the pTEA index group (15.8% vs. 9.7%; *P* = .005) (Table VI). Additionally, after adjusting for patient age, sex, race, and hospital TEA volume in a multivariable logistic regression model, rTEA was independently associated with higher odds of 90-day readmission compared with pTEA (Adjusted OR: 1.55; 95% CI: 0.99 – 2.37; *P* = .048).

**Table VI:** Bivariate analysis of readmission characteristics between primary and revision TEA.

Variable	Overall (n = 1,576)	Primary TEA (n = 1,303)	Revision TEA (n = 273)	*P* value
90-d readmission (%)				**.005**†
Yes	170 (10.8)	127 (9.7)	43 (15.8)	
No	1,406 (89.2)	1,176 (90.3)	230 (84.2)	
Time between surgery and readmission in d (mean ± SD)	51.4 ± 24.7	52.8 ± 25.3	47.3 ± 22.5	.21∗
Number of times readmitted within 90 d (mean ± SD)	1.1 ± 0.4	1.2 ± 0.5	1.0 ± 0.2	**.02**∗
Length of stay during readmission (d) (mean ± SD)	4.7 ± 5.7	4.4 ± 5.0	6.0 ± 8.2	**<.001**∗

*TEA*, total elbow arthroplasty; *SD*, standard deviation.

Bolded *P* values denote a statistically significant value.

∗ Student's t-test.

† Pearson chi-squared test.

Although a higher proportion of rTEA patients were readmitted, pTEA patients were readmitted more times within the 90-day period (*P* = .02). rTEA index patients also stayed longer during their readmission visit than pTEA index patients at 6.0 days vs. 4.4 days (*P* < .001). Notably, the time from surgery to readmission did not differ significantly between primary and revision groups (Table VI). Similarly, no statistically significant differences in readmission characteristics were observed when the cohort was stratified by hospital volume groups alone (Supplemental Table II).

## Discussion

In this study, we found that after accounting for patient and hospital factors, rTEA was independently associated with higher inpatient charges compared to pTEA. Additionally, rTEA patients had a higher likelihood of 90-day readmission and longer inpatient stays compared to pTEA patients. The hospital volume of TEA had a bearing on the total inpatient charges in pTEA. LVHs demonstrated higher total inpatient charges vs. HVHs and MVHs for pTEA but no significant difference was noted in the rTEA cohort.

Although the prevalence of pTEA continues to rise, the rate of increase has slowed considerably, even in the setting of expanding indications.^18^ This is largely due to advances in the medical management of inflammatory arthritis.^2^ Our study demonstrates that, even as the growth rate of TEA in New York State has slowed, hospital charges continue to show an upward trend.^18^ Overall, our findings are consistent with previous studies investigating TEA-associated charges. Day et al,^7^ reported average hospital charges of $51,970 for TEA in the United States in 2007. However, the charges reported were for solely pTEA patients. In our study, we were able to report inpatient charges for both pTEA and rTEA separately due to a distinct code for rTEA introduced in 2013. We found rTEA to be associated with higher inpatient charges, higher LOS, and higher readmission rates. These associations remained significant even after adjusting for patient demographic and institutional factors. This trend is consistent with findings reported in other major joint arthroplasties—where increased procedural complexity, greater likelihood of implant-related challenges, and higher comorbidity burdens contribute to greater complication and readmission rates amongst revision patients.^9^^,^^13^^,^^14^

The secondary goal of this study was to compare charges, costs, and complications from TEA across different hospital procedural volume groups. Interestingly, we observed some differences in charges when the hospitals in our cohort were stratified into high, medium, and low-volume groups for pTEA and rTEA. LVHs had higher overall inpatient charges compared to MVHs and HVHs. Several factors may explain this difference in inpatient charges observed at LVHs compared to MVHs and HVHs for pTEA. One hypothesis is based on the concept of “economies of scale,” in which increased service volume is associated with diminishing unit/service costs, a topic which has previously been demonstrated in total hip arthroplasty (THA) and total knee arthroplasty (TKA).^4^^,^^12^ Blackburn et al reported that higher-volume hospitals performing THA and TKA incurred lower inpatient costs and shorter LOSs, supporting the presence of volume-related cost efficiencies in arthroplasty care.^4^ The underlying factors driving these economies of scale remain unclear and may relate to differences in complication and mortality rates between hospitals, as well as potential advantages in implant procurement from vendors and resource utilization. Consequently, LVHs that do not benefit from these disparities may exhibit higher per-case charges. It is important to note, however, that the impact of higher procedural volume on reducing charges for TEA is likely far more modest than what has been reported for THA and TKA, in part because TEA is performed far less frequently overall, and the resulting differences in case volume between HVHs, MVHs, and LVHs are small. As a result, strong or consistent volume-charge trends for all aspects of the charges were not observed in our cohort.

The average costs for a pTEA at HVHs, MVHs, and LVHs in our study were $17,518, $18,111, and $18,799, respectively, aligning with results by Zhou et al,^21^ who reported an average cost of $16,300 ± $4,000.

Only 58% of patients at LVHs were discharged home without additional care, compared with 61% and 62% at MVHs and HVHs, respectively (*P* = .031). Furthermore, patients at LVHs were, on average, 4 years older than those at MVHs and HVHs (*P* = .003). It is plausible that LVHs may manage a higher proportion of (older) patients with discharge dispositions requiring greater care coordination and involvement from larger care teams, including social workers, pharmacists, and transportation services.

The current study has several limitations. Something not accounted for in this study is that surgeon-specific volume was not analyzed due to insufficient information in SPARCS database. Thus, variations in costs and outcomes based on individual surgeons' expertise cannot be calculated. Secondly, the SPARCS database represents New York State, and thus charges and costs for elbow arthroplasty may not reflect regional variations. Although comorbidity data were available within the SPARCS database, the broad range and variable reporting of diagnostic codes limited our ability to perform meaningful comparisons between pTEA and rTEA groups. Similarly, severity-of-illness information was not available, and ICU admission rates were inconsistently reported across institutions and years, as not all hospitals uniformly code ICU utilization for arthroplasty procedures. These limitations may partly account for observed differences in LOS, charges, and readmission rates. Another limitation common to administrative databases is the potential for coding inaccuracies, which may affect cost and utilization estimates, and we were unable to validate the accuracy of codes used to generate our dataset. However, because the SPARCS database includes only hospitals within New York State—where diagnostic and procedural coding is regulated and periodically audited by the Department of Health—reporting practices are relatively standardized across institutions, and any resulting inaccuracies are likely minimal. Furthermore, hospitals typically receive only a portion of the charges they bill, meaning the reported charges tend to overestimate the actual payments. The payments hospitals receive can vary significantly based on factors such as patient demographics, type of insurance, and level of care provided. For example, socioeconomic and census data were factors not included in our analysis, although these factors may influence inpatient charges. However, the impact of socioeconomic limitations is partially offset by the inclusion of a large and diverse patient population in the study. Additionally, due to a wide variety of indications, it was difficult to assess the impact of specific indications for revision surgeries on charges, which severely limits the ability to assess why prices were different. Similarly, there is no way to determine what proportion of the 273 patients in our revision cohort is simultaneously accounted for in the pTEA cohort. Furthermore, our threshold for categorizing hospitals as high-volume was intentionally set lower than conventional definitions used in hip or knee arthroplasty literature to account for the relative rarity of TEA. While this approach allowed for a more balanced distribution of cases across volume groups, it may limit the generalizability of our findings to other arthroplasty types and should be interpreted cautiously. This lower threshold could attenuate or obscure true volume–cost relationships that might be more apparent with larger procedural datasets. Lastly, 38 hospitals, collectively comprising 128 TEAs, were excluded from the analysis due to meager TEA volume, which may introduce bias to the study cohort. Despite these limitations, there is a paucity of literature on the inpatient charges associated with TEA and, even more so, on the impact of hospital volume on inpatient charges. Our study provides valuable insights into these areas and lays a foundation for further research.

## Conclusion

rTEA is associated with longer inpatient stay, higher inpatient charges, and greater readmission rates compared to pTEA. pTEAs in low-volume hospitals are associated with higher total charges compared to medium- and high-volume hospitals. These findings provide valuable insights for hospital administrators and public health officials aiming to create effective strategies to manage costs and combat the growing burden of healthcare expenses in the United States.

## Disclaimers:

Funding: No funding was disclosed by the authors.

Conflicts of interest: Mandeep S. Virk is a paid consultant for Exactech, Inc. All the other authors, their immediate families, and any research foundations with which they are affiliated have not received any financial payments or other benefits from any commercial entity related to the subject of this article.
