# Supplementary material for: Comparison of inpatient charges and costs between revision and primary total elbow arthroplasty in the New York state
Source: JSES Rev Rep Tech. 2025 Dec 24;6(2):100648. doi: 10.1016/j.xrrt.2025.100648 (PMC12887384; doi:10.1016/j.xrrt.2025.100648)
Supplement: Supplementary Tables [file mmc1.docx]

**Supplementary Table I: Inpatient Charges Analysis for Primary and Revision TEA in United States Dollars**

| **Variable** | **Overall (n=1,576)** | **Primary TEA (n=1,303)** | **Revision TEA (n=273)** | ***P* value** |
| --- | --- | --- | --- | --- |
| Accommodation Charges **(**Median [IQR]) | 10,500.00 [5,113.50, 21,017.00] | 10,200.00 [5,278.00, 19,871.64] | 12,650.00 [4,700.00, 25,680.00] | 0.84^§^ |
| Ancillary Charges **(**Median [IQR]) | 40,443.72 [26,139.07, 61,280.19] | 40,383.30 [27,062.89, 59,728.30] | 40,663.07 [21,125.55, 76,137.00] | 0.26^§^ |
| Total Charges **(**Median [IQR]) | 53,304.26 [35,412.34, 86,013.67] | 52,937.13 [36,250.35, 82,410.64] | 56,110.73 [28,865.55, 101,748.35] | 0.26^§^ |

*TEA, total elbow arthroplasty*

*IQR, Interquartile Range*

*Bolded P-values denote a statistically significant value.*

*§ Mann-Whitney U test*

**Supplementary Table II : Bivariate Analysis of Readmission Characteristics by Hospital Volume Among TEA Patients**

| **Variable** | **Overall (n=1,576)** | **High (n=863)** | **Medium (n=405)** | **Low (n=308)** | ***P* value** |
| --- | --- | --- | --- | --- | --- |
| **90-day readmission (%)** |  |  |  |  | 0.08*^†^* |
| No | 1,406 (89.2) | 775 (89.8) | 367 (90.6) | 264 (85.7) |  |
| Yes | 170 (10.8) | 88 (10.2) | 38 (9.4) | 44 (14.3) |  |
| **Time between surgery and readmission (days)**  **(mean ± SD)** | 51.4 ± 24.65 | 51.9 ± 24.5 | 52.0 ± 25.7 | 49.9 ± 24.5 | 0.90 *^*^* |
| **Number of times readmitted within 90 days**  **(mean ± SD)** | 1.1 ± 0.4 | 1.1 ± 0.3 | 1.2 ± 0.5 | 1.2 ± 0.4 | 0.56 *^*^* |
| **Length of stay (days)**  **(mean ± SD)** | 4.7 ± 5.7 | 4.4 ± 5.4 | 4.9 ± 6.4 | 5.0 ± 5.6 | 0.18 *^*^* |

*TEA, total elbow arthroplasty*

*Bolded P-values denote a statistically significant value.*

*SD, Standard deviation*

** Student’s t-test*

*† Pearson chi-squared test*
